# X-ray crystalographic data, absolute configuration, and anticholinesterase effect of dihydromyricitrin 3-*O*-rhamnoside

**DOI:** 10.1038/s41598-022-23240-7

**Published:** 2022-11-01

**Authors:** Mahmoud Fahmi Elsebai, Hazem A. Ghabbour, Ramin Ekhteiari Salmas, Ilkay Erdogan Orhan, Fatma Sezer Senol Deniz

**Affiliations:** 1 Department of Pharmacognosy, Faculty of Pharmacy, Mansoura University, Mansoura, 35516 Egypt; 2 Department of Medicinal Chemistry, Faculty of Pharmacy, Mansoura University, Mansoura, 35516 Egypt; 3 Department of Chemistry, Britannia House, King’s College London, London, UK; 4 Department of Pharmacognosy, Faculty of Pharmacy, Gazi University, 06330 Ankara, Türkiye

**Keywords:** Chemical biology, Drug discovery, Plant sciences

## Abstract

Based on our continuous effort to investigate chemistry and biology of the plant secondary metabolites, we were able to isolate a glycosidal flavonoid **1** from the Wild Egyptian Artichoke. The activity of dihydromyricetin 3-*O*-rhamnoside (sin. dihydromyricitrin, ampelopsin 3-*O*-rhamnoside) (**1**) against acetylcholinesterase (AChE) and butyrylcholinesterase (BChE); its absolute configuration using X-ray crystallography were determined for the first time. Inhibitory activity of **1** against AChE and BChE enzymes were determined using a slightly modified version of Ellman’s method. Compound **1** was revealed to have a potent inhibition against acetylcholinesterase (AChE) and butyrylcholinesterase (BChE) with IC_50_ values of 0.070 ± 0.008 and 0.071 ± 0.004 mM, respectively, where IC_50_ values of the reference drug (galanthamine) were 0.023 ± 0.15 and 0.047 ± 0.91 mM. Compound **1** could be a promising molecule against Alzheimer’s disease.

## Introduction

**Figure 1:**
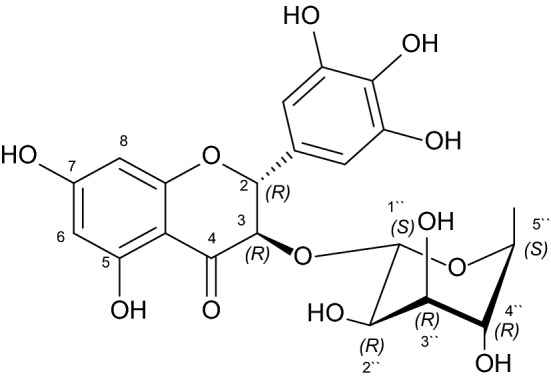
Structure of 2*R*, 3*R*, 1´´*S*, 2´´*R*, 3´´*R*, 4´´*R*, 5´´*S* dihydromyricetin 3-*O*-rhamnoside (**1**).

In many countries, artichoke is a traditionally consumed vegetable and is used as a leaf plant to produce extracts for dietary and medicinal applications. The artichoke plants and their constituents exhibit diverse and important pharmacological effects and health benefits^1–4^. The phytochemical investigation of the artichoke leaf extract resulted in the isolation of compound **1** (Fig. 1). The crystals of **1** were obtained using a mixture of chloroform and methanol and its crystallographic data was obtained which enabled us to determine the absolute configuration. Simulating the chemical molecular interactions formed between ligands and active-site amino acids of proteins brings us to a level of understanding that why ligands would reveal different biochemical characteristics including inhibitory effects on some certain targets and how this would vary between ligands and proteins. With a clear understanding about the atomic interactions triggering compounds to inhibit enzymes would lead us to the development of potential inhibitor compounds. In this study the results of the docking simulations carried out on complexes of AChE and BChE with compound **1** help us determine those amino acids playing a key role inside the ligand-binding domains—and the dominant poses that would be observed.

## Results and discussion

The methanolic extract of the leaves of artichoke plant yielded compound **1** after fractionation and repeated chromatographic separations on silica gel column as in references^1,5^.

**Figure 2:**
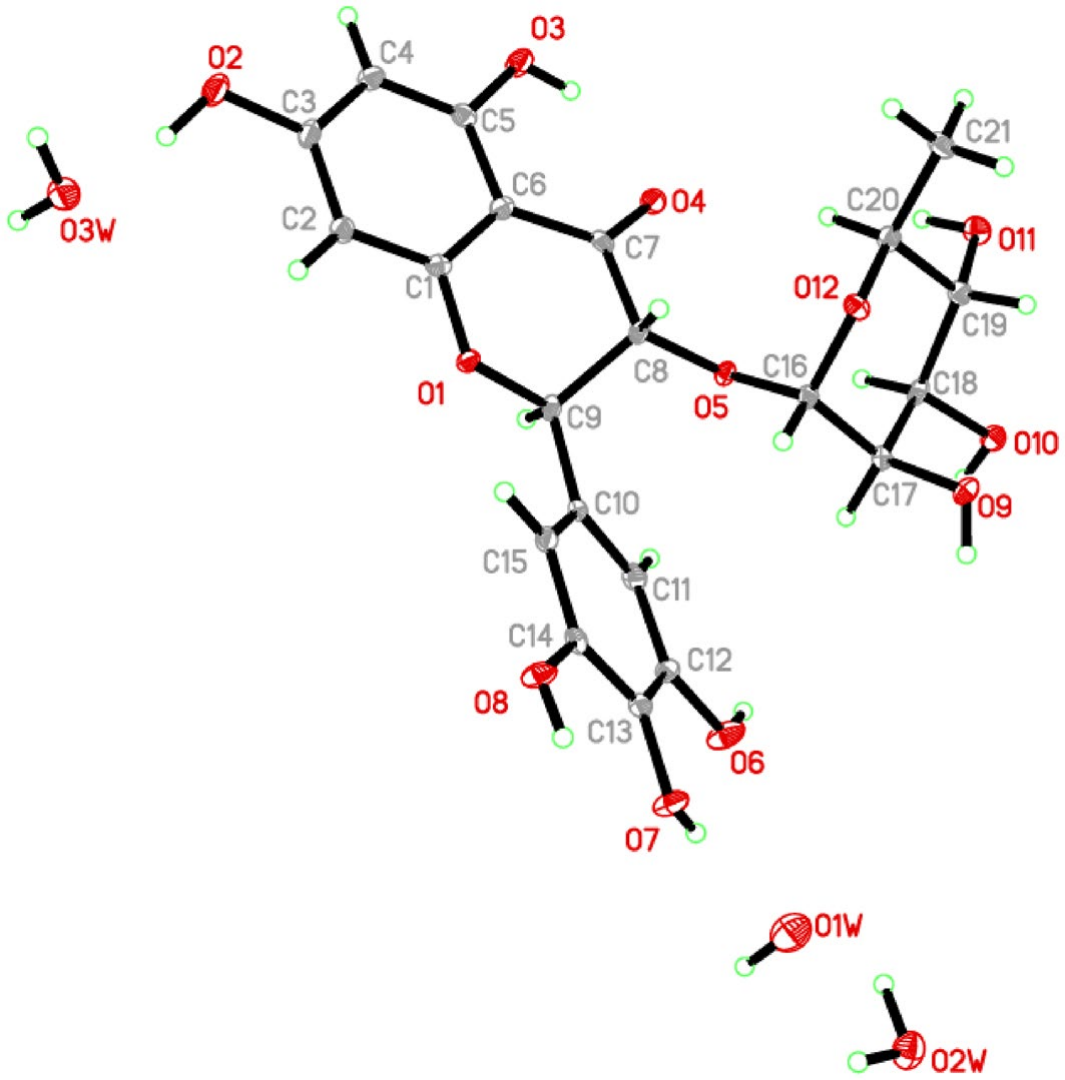
ORTEP of compound **1**. Non-H atoms have their displacement ellipsoids depicted at a 40% probability level.

Needle crystals of compound **1** were formed from a solvent mixture of chloroform and methanol. The structure of **1** was confirmed by the single-crystal X-ray crystallographic measurements (Fig. 2) and accurate mass measurements (measured *m*/*z* 465.1030 (M–H^+^); calculated 465.1033(M–H^+^)) and the absolute configuration of the seven chiral centers was determined to be 2*R*, 3*R*, 1´´*S*, 2´´*R*, 3´´*R*, 4´´*R*, and 5´´*S*.

**Figure 3:**
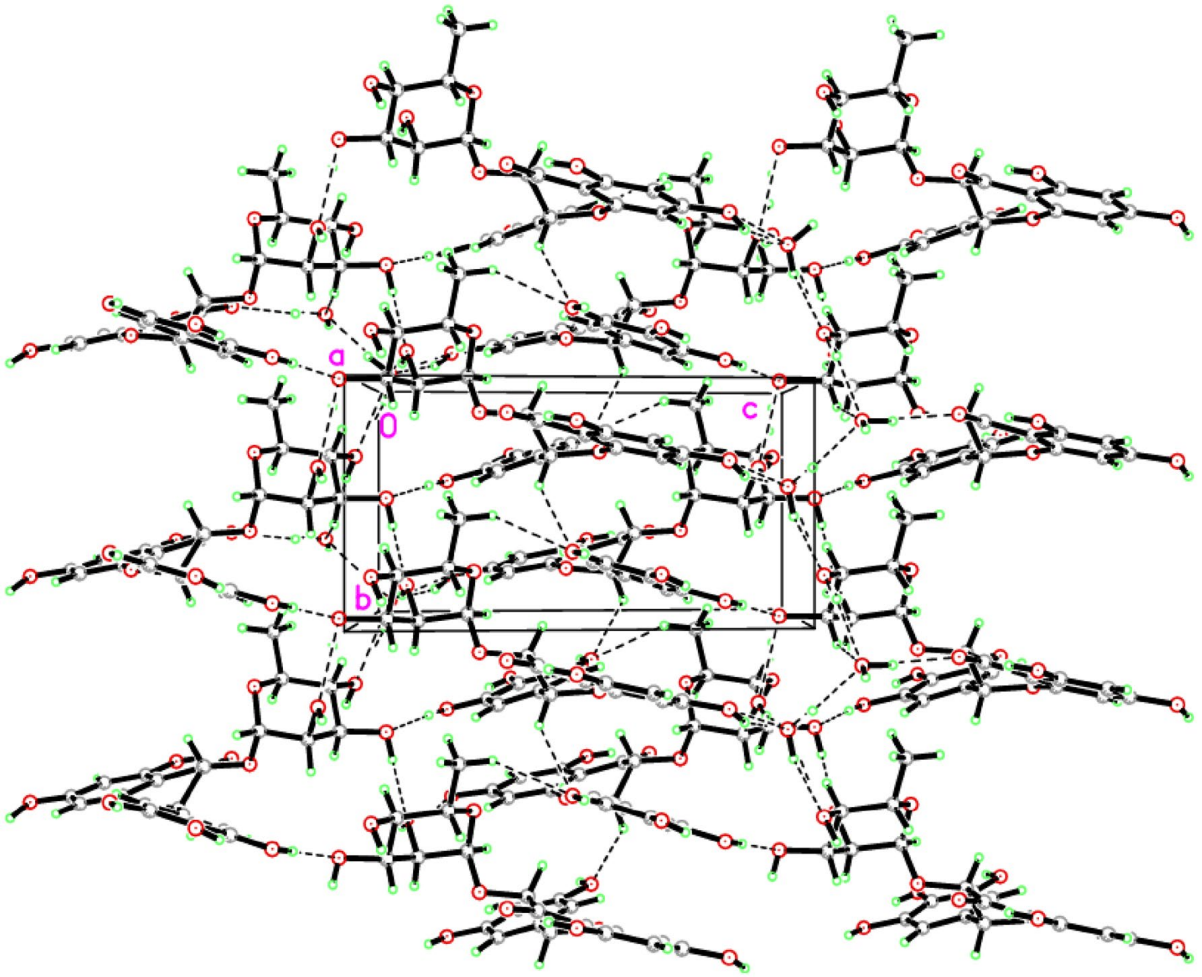
Compound **1**’s molecular docking revealed a network of H bonds, which are shown as dashed lines.

In the current work, inhibitory effect of compound **1** against AChE and BChE was assayed, which was found to possess a marked inhibition with IC_50_ values of 0.070 ± 0.008 and 0.071 ± 0.004 mM, respectively. IC_50_ values of the reference drug (galanthamine) towards AChE and BChE were revealed to be 0.023 ± 0.15 and 0.047 ± 0.91 mM. The chemical interactions of dihydromyricetin 3-*O*-rhamnoside inside AChE and BChE were simulated using IFD method—by which it can be figured out how the ligand can stick to the binding domains of the proteins and which amino acids have mostly contributed to the interactions. This method is able to generate multiple poses for a single ligand; thus, the ligand-binding energy would vary from pose to pose—following our understanding that ligands inside the binding domains are dynamically stable (Fig. 3).

**Figure 4:**
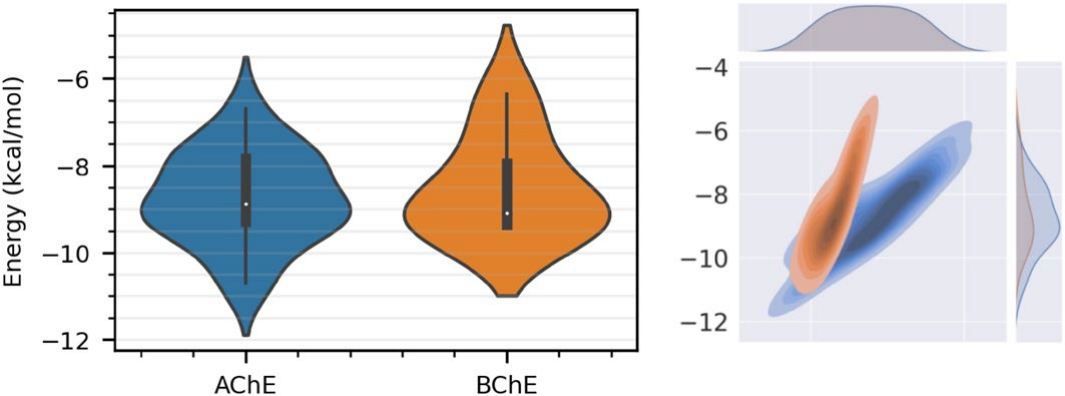
The IFD scores of compound **1** in different poses inside AChE and BChE – the minimum, maximum, first quartile, median, and third quartile values are presented (left panel). Probability density function of the IFD scores between the two systems are compared using KDE method (right panel). The AChE complex was represented with more varieties of the poses which lead to diverse binding energies, compared to the BChE complex. The lowest binding energies were calculated around − 12 and − 10 kcal/mol for the AChE and BChE complexes, respectively, demonstrating that AChE is more favourite for compound **1**.

The distributions of the binding energies of the compound inside both AChE and BChE were presented using box plot along with their frequencies in Fig. 4 (the graph in the left panel). The plots present the minimum, maximum, first quartile, median, and third quartile values of the binding energies. The lowest binding energies of the compound were calculated to be around − 12 and − 11 kcal/mol inside AChE and BChE targets, respectively. Both the proteins revealed nearly the same median value, − 8 kcal/mol—by which it would be understand that the compound can sticks to the proteins through favorable interactions. If the binding energies are compared in the two proteins, AChE has been found more accommodating for interaction with the compound. The graph in the right panel in Fig. 4 corresponds to kernel density estimate (KDE) method that measures the probability density function (PDF), yielding the probability that the docking poses fall within specific ranges of binding energies. There are not significant differences between the PDF of the AChE and BChE complexes, but they are not equitable. The portions ranging from − 8 to − 10 kcal/mol have been more converged—however, the distribution belonging to AChE (labeled by red color) has been skewed to the right.

**Figure 5:**
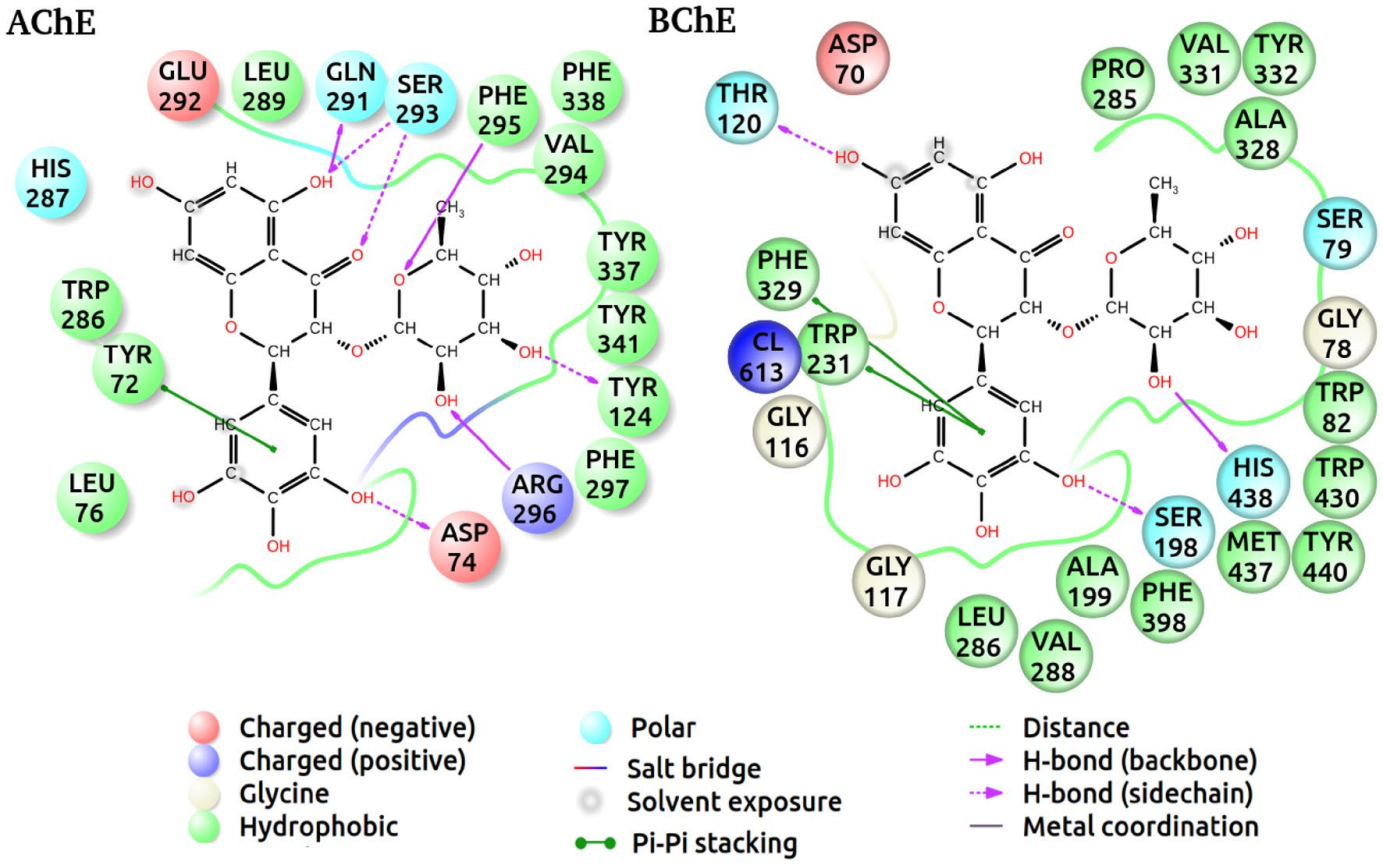
2D diagrams of compound **1** inside AChE and BChE. The important amino acids surrounding the ligand are presented with different colors, which correspond to their chemical properties. The polar and nonpolar interactions formed between the atoms of the ligand and the amino acids are shown differently (see the legend). The poses were generated from the systems with the lowest binding energies (top scores), which represent the position of the ligand for each complex—however the interactions would vary between the complexes.

Figure 5 shows the representative poses of the compound inside the AChE and BChE, generated through the top-score poses. What we can understand from the 2D diagrams are the potential interactions (including the polar and nonpolar bonds) formed between the compound and the neighboring amino acids. Additionally, these can create a clearer picture of the roles and level of contribution of each amino acid inside the binding domain, which would lead us to a much deeper understanding of the inhibition mechanism. Regarding the AChE complex, the polar interactions are more dominant, to which amino acids Asp74, Arg296, Tye124, Phe295, Ser293 and Gln291 contributed. Tyr72 has been found the only amino acid forming a Pi–Pi stacking interaction with the aromatic ring of the compound.

The compound in AChE unlike that in BChE almost equally formed proportion of both hydrogen bonds and hydrophobic interactions with the amino acids. The diagrams suggest that amino acids Ser198 (sidechain) and His438 (backbone) in BChE correspond to amino acids Asp74 (sidechain) and Arg296 (backbone) in BChE, respectively, which have contributed to forming hydrogen bonds with the hydroxyl groups of the ligand. It has been found that both amino acids Phe329 and Trp231 from BChE participated in Pi–Pi stacking interactions with the aromatic ring of the compound—analogous to Tyr72 in AChE.

### Crystal data of compound 1

The molecular structure of **1** was crystallized in the Monoclinic, *P*2_1_, *a* = 12.2665 (3) Å, *b* = 6.9450 (2) Å,* c* = 12.9980 (3) Å, °, *β* = 97.336 (1)°, *V* = 1098.25 (5) Å^3^, *Z* = 2. In the title molecule, C_21_H_27_O_15,_ the crystallographic data and refinement information are presented in the supplementary data (Table s1). Table 1 shows the specified bond lengths and angles. As seen in Fig. 2, the asymmetric unit has one independent molecule in addition to three water molecules. The bonds parameters such as length and angle are all within acceptable limits^6^. Many intermolecular hydrogen bonds connect molecules in the crystal packing (Fig. 3; Table 2).

**Table 1:** Specified bond lengths and angles (Å, °).

O1–C1	1.364 (3)	O7–C13	1.374 (3)
O1–C9	1.451 (3)	O8–C14	1.371 (3)
O2–C3	1.357 (3)	O9–C17	1.429 (3)
O3–C5	1.356 (3)	O10–C18	1.435 (3)
O4–C7	1.242 (3)	O11–C19	1.438 (3)
O5–C8	1.418 (3)	O12–C16	1.409 (3)
O5–C16	1.420 (3)	O12–C20	1.440 (3)
O6–C12	1.372 (3)		
C1–O1–C9	116.06 (19)	O6–C12–C13	116.5 (2)
C8–O5–C16	113.7 (2)	O7–C13–C14	117.0 (2)
C16–O12–C20	113.9 (2)	O7–C13–C12	123.8 (2)
O1–C1–C2	116.8 (2)	O8–C14–C13	120.7 (2)
O1–C1–C6	121.3 (2)	O8–C14–C15	118.3 (2)
O2–C3–C4	116.5 (2)	O5–C16–O12	112.0 (2)
O2–C3–C2	121.9 (2)	O5–C16–C17	106.2 (2)
O3–C5–C4	118.8 (2)	O12–C16–C17	111.0 (2)
O3–C5–C6	119.7 (2)	O9–C17–C16	107.5 (2)
O4–C7–C8	122.6 (2)	O9–C17–C18	110.6 (2)
O4–C7–C6	123.5 (2)	O10–C18–C17	111.3 (2)
O5–C8–C9	112.4 (2)	O10–C18–C19	107.8 (2)
O5–C8–C7	111.0 (2)	O11–C19–C20	109.3 (2)
O1–C9–C8	107.4 (2)	O11–C19–C18	110.7 (2)
O1–C9–C10	108.4 (2)	O12–C20–C19	110.6 (2)
O6–C12–C11	123.1 (2)	O12–C20–C21	107.1 (2)

**Table 2:** Hydrogen-bonds geometry (Å, °).

*D*–H···*A*	*D*–H	H···*A*	*D*···*A*	*D*–H···*A*
O10–H1O0···O9^i^	0.84 (6)	1.82 (6)	2.648 (3)	166 (4)
O11–H1O1···O2W^ii^	0.88 (5)	2.05 (5)	2.886 (3)	159 (4)
O3W–H1W3···O11^iii^	0.85 (6)	1.95 (6)	2.759 (3)	160 (5)
O2W–H2W2···O1W	1.01 (5)	1.93 (5)	2.822 (3)	146 (5)
O9–H1O9···O3W^iv^	0.82 (5)	1.96 (5)	2.754 (3)	164 (4)
O2W–H1W2···O4^v^	0.96 (4)	1.96 (4)	2.897 (3)	166 (4)
O2–H1O2···O3W	0.93 (5)	1.85 (5)	2.739 (3)	160 (5)
O8–H1O8···O3^v^	0.81 (5)	2.00 (5)	2.763 (3)	157 (4)
O7–H1O7···O1W	0.74 (4)	2.20 (4)	2.925 (3)	166 (4)
O1W–H1W1···O2v^i^	0.92 (10)	2.17 (9)	3.013 (4)	152 (6)
O6–H1O6···O10^i^	0.77 (5)	1.94 (5)	2.710 (3)	177 (5)
O3–H1O3···O4	0.81 (4)	1.92 (4)	2.624 (3)	145 (4)
O3W–H2W3···O2W^vii^	0.92 (5)	1.97 (5)	2.886 (3)	173 (5)
C9–H9A···O8^vi^	1.00	2.53	3.350 (3)	139.00
C18–H18A···O2W^ii^	1.00	2.54	3.354 (3)	139.00
C20–H20A···O4	1.00	2.49	3.453 (3)	160.00
C21–H21B···O8^iv^	0.98	2.53	3.385 (3)	146.00

## Methods

### Plant material, extraction, and isolation

See references^1,5,6^. The plant (*Cynara cardunculus* L. var. *sylvestris* (Lam.) Fiori) was identified by Prof. Dr. Ahmed R. Atawia. A voucher specimen with the code “2007-artich-Sinai” was deposited in the Department of Horticulture, Faculty of Agriculture, Benha University, Egypt. The plant name has been checked with http://www.theplantlist.org. The permission to collect artichoke plant and research on it was approved by the Ethical Committee of Research at the Faculty of Pharmacy, Mansoura University, Egypt (code number 2014/71) and was consistent with GCP guidelines and the applicable regulatory requirements.

### X-ray crystallography

As in reference^7^ Molecule **1** was produced as single crystals by gradual evaporation of the pure compound in a solvent combination of chloroform and methanol at ambient temperature. Data were acquired using a Bruker APEX-II D8 Venture area diffractometer with graphite monochromatic Cu K radiation, = 1.54178 at 296 (2) K. Bruker SAINT performed cell refinement and data reduction. The structure was solved using SHELXT^9,10^. Full-matrix least-squares approaches using anisotropic thermal data for nonhydrogen atoms on F were used for the final refinement. The Cambridge Crystallographic Data Centre’s CCDC 2161839, which provides supplemental crystallographic data for this chemical, may be downloaded for free at https://www.ccdc.cam.ac.uk/datarequest/cif.

### Cholinesterase inhibition assays

Inhibitory activities of compound **1** against AChE and BChE enzymes were determined using the modified spectrophotometric method of Ellman procedure^8^. The enzyme sources were electric eel acetylcholinesterase (Type-VI-S, EC 3.1.1.7, Sigma) and horse serum butyrylcholinesterase (EC 3.1.1.8, Sigma), while the reaction substrates were acetylthiocholine iodide and butyrylthiocholine chloride (Sigma, St. Louis, MO, USA). Our previous paper thoroughly explains the cholinesterase enzyme inhibition experiments we used in the current study^9^.The IC_50_ values of compound 1 were calculated via GraphPad Prism 6.01.

### Ligand–protein docking simulations

Crystal structures of AChE and BChE were downloaded from Protein Data Bank (PDB)^10^—their PDB codes are 4EY7^11^ and 5DYW^12^, respectively. Missing atoms in the backbones and sidechains were added, and the broken and disulfide bonds in the amino acids were fixed by Protein Preparation Wizard of the maestro of Schrödinger software^13,14^. The PROPKA tool^15^ was used for predicting the ionization of the systems and evolving it based on biological pH. The energies between the atoms are minimized to remove any potential clashes between the atoms, using OPLS3 force field through the classical minimization. The structure of the ligand was sketched and minimized in biological pH. Protein–ligand simulation was carried out using induced-Fit docking (IFD) method^16–18^, by which more flexibility is given to both ligand and the amino acids in the binding domain to refine themselves during the simulation. The coordinates of the co-crystal ligands inside the proteins were used to understand where to place the docking simulation box.

## Conclusion

The plants are prolific producers of natural products. Natural products are lead drugs for treatment of many diseases. Many of the antibiotics are essentially natural products such as erythromycins, aminoglycosides and the antitumor antibiotics. Additionally, there is a huge number of their derivatives which were semisynthesized from the lead drugs of natural products such penicillins and cephalosporins. Compound **1** showed promising activity against acetylcholinesterase (AChE) and butyrylcholinesterase (BChE). These enzymes were associated with pathology of Alzheimer’s disease. Additionally, the absolute configuration of compound **1** were determined for the first time.

## Supplementary Information

Supplementary Information.

## Data Availability

The datasets generated and/or analyzed during the current study are available in The Cambridge Crystallographic Data Centre's CCDC 2161839; it may be downloaded for free at https://www.ccdc.cam.ac.uk/datarequest/cif. https://www.ccdc.cam.ac.uk/structures/Search?access=referee&ccdc=2161839&Author=Hazem+Ghabbour.
