# Supplementary material for: X-ray crystalographic data, absolute configuration, and anticholinesterase effect of dihydromyricitrin 3-O-rhamnoside
Source: Sci Rep. 2022 Nov 1;12:18351. doi: 10.1038/s41598-022-23240-7 (PMC9626498; doi:10.1038/s41598-022-23240-7)
Supplement: Supplementary file 1 — Supplementary Information. [file 41598_2022_23240_MOESM1_ESM.docx]

**X-Ray Crystalographic Data, Absolute Configuration, and Anticholinesterase Effect of Dihydromyricitrin 3-*O*-rhamnoside**

Mahmoud Fahmi Elsebai^a,^*, Hazem A. Ghabbour^b^, Ramin Ekhteiari Salmas^c^,

Ilkay Erdogan Orhan^d^, Fatma Sezer Senol Deniz^d^

^a^Department of Pharmacognosy, Faculty of Pharmacy, Mansoura University, 35516, Mansoura, Egypt.

^b^Department of Medicinal Chemistry, Faculty of Pharmacy, Mansoura University, Mansoura 35516, Egypt.

^c^Department of Chemistry, Britannia House, King’s College London, UK.

^d^Department of Pharmacognosy, Faculty of Pharmacy, Gazi University, 06330 Ankara, Türkiye.

*Corresponding author:

Mahmoud F. Elsebai, E-mail: [elsebai72@yahoo.com](mailto:elsebai72@yahoo.com); [elsebai72@mans.edu.eg](mailto:elsebai72@mans.edu.eg)

**Keywords:** Dihydro-Myricetin 3-*O*-rhamnoside; ampelopsin 3-*O*-rhamnoside; acetylcholinesterase; butyrylcholinesterase; docking simulation.

Table s1. Experimental details

| **Crystal data** | |
| --- | --- |
| Chemical formula | [C_21_H_27_O_15_](file:///C:\C:\Users\pc\Downloads\gohr_2%20_chemical_formula_sum) |
| *M*r | [519.42](file:///C:\C:\Users\pc\Downloads\gohr_2%20_chemical_formula_weight) |
| Crystal system, space group | [Monoclinic](file:///C:\C:\Users\pc\Downloads\gohr_2%20_space_group_crystal_system), [*P*2_1_](file:///C:\C:\Users\pc\Downloads\gohr_2%20_space_group_name_H-M_alt) |
| Temperature (K) | [296](file:///C:\C:\Users\pc\Downloads\gohr_2%20_cell_measurement_temperature) |
| *a*, *b*, *c* (Å) | [12.2665 (3)](file:///C:\C:\Users\pc\Downloads\gohr_2%20_cell_length_a), [6.9450 (2)](file:///C:\C:\Users\pc\Downloads\gohr_2%20_cell_length_b), [12.9980 (3)](file:///C:\C:\Users\pc\Downloads\gohr_2%20_cell_length_c) |
| *β* (°) | [97.336 (1)](file:///C:\C:\Users\pc\Downloads\gohr_2%20_cell_angle_beta) |
| V (Å3) | [1098.25 (5)](file:///C:\C:\Users\pc\Downloads\gohr_2%20_cell_volume) |
| *Z* | [2](file:///C:\C:\Users\pc\Downloads\gohr_2%20_cell_formula_units_Z) |
| Radiation type | [Cu *K*α](file:///C:\C:\Users\pc\Downloads\gohr_2%20_diffrn_radiation_type) |
| µ (mm^−1^) | [1.18](file:///C:\C:\Users\pc\Downloads\gohr_2%20_exptl_absorpt_coefficient_mu) |
| Crystal size (mm) | [0.42](file:///C:\C:\Users\pc\Downloads\gohr_2%20_exptl_crystal_size_max) × [0.16](file:///C:\C:\Users\pc\Downloads\gohr_2%20_exptl_crystal_size_mid) × [0.07](file:///C:\C:\Users\pc\Downloads\gohr_2%20_exptl_crystal_size_min) |
| **Data collection** | |
| Diffractometer | Bruker APEX-II D8 venture diffractometer |
| Absorption correction | Multi-scan SADABS Bruker 2018 |
| Tmin, Tmax | [0.934](file:///C:\C:\Users\pc\Downloads\gohr_2%20_exptl_absorpt_correction_T_min), [0.965](file:///C:\C:\Users\pc\Downloads\gohr_2%20_exptl_absorpt_correction_T_max) |
| No. of measured, independent and observed [I > 2σ(I)] reflections | [14444](file:///C:\C:\Users\pc\Downloads\gohr_2%20_diffrn_reflns_number), [3755](file:///C:\C:\Users\pc\Downloads\gohr_2%20_reflns_number_total), [3511](file:///C:\C:\Users\pc\Downloads\gohr_2%20_reflns_number_gt) |
| R_int_ | [0.049](file:///C:\C:\Users\pc\Downloads\gohr_2%20_diffrn_reflns_av_R_equivalents) |
| **Refinement** | |
| R[*F^2^* > 2σ( *F^2^*)], wR( *F^2^*), S | [0.035](file:///C:\C:\Users\pc\Downloads\gohr_2%20_refine_ls_R_factor_gt), [0.082](file:///C:\C:\Users\pc\Downloads\gohr_2%20_refine_ls_wR_factor_ref), [1.08](file:///C:\C:\Users\pc\Downloads\gohr_2%20_refine_ls_goodness_of_fit_ref) |
| No. of reflections | [3755](file:///C:\C:\Users\pc\Downloads\gohr_2%20_refine_ls_number_reflns) |
| No. of parameters | [377](file:///C:\C:\Users\pc\Downloads\gohr_2%20_refine_ls_number_parameters) |
| No. of restraints | [1](file:///C:\C:\Users\pc\Downloads\gohr_2%20_refine_ls_number_restraints) |
| H-atom treatment | [H atoms treated by a mixture of independent and constrained refinement](file:///C:\C:\Users\pc\Downloads\a%20_refine_ls_hydrogen_treatment) |
| Δρ_max_, Δρ_min_ (e Å^−3^) | [0.27](file:///C:\C:\Users\pc\Downloads\gohr_2%20_refine_diff_density_max), [−0.34](file:///C:\C:\Users\pc\Downloads\gohr_2%20_refine_diff_density_min) |
| Absolute structure | Flack x determined using 1419 quotients [(I+)-(I-)]/[(I+)+(I-)] (Parsons, Flack and Wagner, Acta Cryst. B69 (2013) 249-259). |
| Absolute structure parameter | [−](file:///C:\C:\Users\pc\Downloads\gohr_2%20_refine_ls_abs_structure_Flack)0.05 (10) |
